# Spectroscopic Manifestations and Implications for Catalysis of Quasi‐d^10^ Configurations in Formal Gold(III) Complexes

**DOI:** 10.1002/anie.202215523

**Published:** 2022-12-12

**Authors:** Evgeniya A. Trifonova, Isaac F. Leach, Winfried B. de Haas, Remco W. A. Havenith, Moniek Tromp, Johannes E. M. N. Klein

**Affiliations:** ^1^ Molecular Inorganic Chemistry Stratingh Institute for Chemistry University of Groningen Nijenborgh 4 9747 AG Groningen The Netherlands; ^2^ Zernike Institute for Advanced Materials University of Groningen Nijenborgh 4 9747 AG Groningen The Netherlands; ^3^ Ghent Quantum Chemistry Group Department of Chemistry Ghent University 9000 Gent Belgium

**Keywords:** Catalysis, Computational Chemistry, Covalent Bonding, Gold, Oxidation States

## Abstract

Several gold +I and +III complexes are investigated computationally and spectroscopically, focusing on the d‐configuration and physical oxidation state of the metal center. Density functional theory calculations reveal the non‐negligible electron‐sharing covalent character of the metal‐to‐ligand σ‐bonding framework. The bonding of gold(III) is shown to be isoelectronic to the formal Cu^III^ complex [Cu(CF_3_)_4_]^1−^, in which the metal center tries to populate its formally unoccupied 3d_x2‐y2_ orbital via σ‐bonding, leading to a reduced d^10^ Cu^I^ description. However, Au L_3_‐edge X‐ray absorption spectroscopy reveals excitation into the d‐orbital of the Au^III^ species is still possible, showing that a genuine d^10^ configuration is not achieved. We also find an increased electron‐sharing nature of the σ‐bonds in the Au^I^ species, relative to their Ag^I^ and Cu^I^ analogues, due to the low‐lying 6s orbital. We propose that gold +I and +III complexes form similar bonds with substrates, owing primarily to participation of the 5d_x2‐y2_ or 6s orbital, respectively, in bonding, indicating why Au^I^ and Au^III^ complexes often have similar reactivity.

## Graphical abstract

Through the interplay of X‐ray spectroscopy and computational analysis, we show that gold +I and +III complexes, despite their physically distinct electronic structures, tend to activate substrates to similar extents. We focus in particular on the role of electron‐sharing covalency of the metal‐ligand bonds and provide an explanation for the often‐similar reactivities observed for gold complexes in these physical oxidation states.

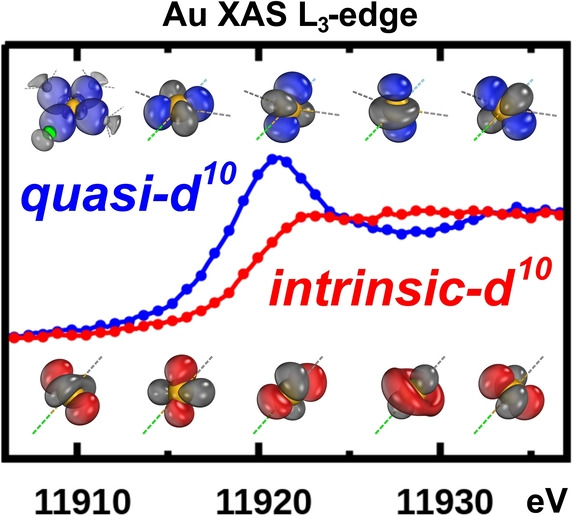

## Introduction

The field of gold catalysis initially emphasized the use of gold(I) and gold(III) species by creatively exploiting their Lewis acidic properties and π‐activation ability e.g. to functionalize unsaturated substrates.^[1]^ A curiosity that remains in this research field is why many transformations are known to be promoted similarly well by either gold(I) or gold(III) species.^[2]^ Although the redox chemistry of gold has been known for several decades,^[3]^ it was not until more recently that it drew considerable attention. The first well‐characterized oxidative addition to a single gold(I) center, forming gold(III), was reported in 2014 by Bourissou and co‐workers^[4]^ and it exemplifies the fact that the interchange between gold's oxidation states (***OS*** s) is becoming more relevant,^[5]^ and more recent developments now lead to the inclusion of oxidation state changes between gold(I) and gold(III) into catalytic cycles.^[6]^

IUPAC fairly recently defined the ***OS*** of an atom in a molecule as the “charge after ionic approximation of its bonds”,^[7]^ but it is a long‐standing concept in chemical theory that is used to rationalize and predict reaction outcomes. Elucidating the role gold's ***OS*** s play in reactivity may therefore guide future developments. On this quest, we may gain insight and inspiration from other coinage metals. Isoelectronic to gold(III), the electronic structure of formal d^8^ copper(III) complexes, such as [Cu(CF_3_)_4_]^1−^, has been the subject of much debate.^[8]^ Snyder first raised the question of whether we had better consider [Cu(CF_3_)_4_]^1−^ as containing a d^10^ copper(I) center.^[8a]^ Total d‐orbital populations approaching ten in quantum mechanical calculations were reported, ascribed to a high degree of electron‐sharing covalency in the metal‐ligand σ‐bonding framework.[^8a^, ^8c^] The observation of this increased covalency is consistent with measured and simulated Cu X‐ray absorption spectra (XAS).[^8f^, ^8g^, ^9^] While this has been used to argue that all formal copper(III) centers are in fact better described as d^10^ copper(I),^[8g]^ a more recent in‐depth study (employing both Cu XAS and Cu K*β* valence‐to‐core X‐ray emission spectroscopy) has provided “strong evidence for the presence of a low‐spin d^8^ Cu^III^” center in [Cu(CF_3_)_4_]^1−^.^[9]^ We recently revisited the electronic structure of [Cu(CF_3_)_4_]^1−^ and found, using an Intrinsic Bond Orbital (IBO) analysis^[10]^ based on Density Functional Theory (DFT) calculations, that the metal center can indeed be described as having eight intrinsic d‐electrons.^[11]^ This is in close agreement with the work of Salvador, Head‐Gordon and co‐workers, who used similar methods.^[12]^ In accordance with Snyder's original proposition, and the interpretation of the spectroscopic data, another two electrons worth of density are recovered by the metal via electron‐sharing covalent σ‐bonds with the ligand. However, we note here that this recovery (or “charge self‐regulation”)^[13]^ is incomplete, as a genuine d^10^ configuration is never obtained. We concluded that the tension between the intrinsic d^*8*^ configuration of copper(III) and the summed (bonding+non‐bonding) d‐electron populations can be resolved by labelling this bonding scenario as a quasi‐d^10^ configuration of the metal.^[11]^ This description conveniently maintains the distinction to classical d^10^ copper(I) complexes such as [Cu(CF_3_)_2_]^1−^, whose 3d‐orbitals are fully occupied.

Pivoting back to gold, we may therefore ask:

- is a quasi‐d^10^ configuration an appropriate description of formal gold(III) complexes?
- how different are the spectroscopic signatures of typical gold(I) and gold(III) complexes?
- do (a) and (b) carry implications for the roles of gold(I) vs. gold(III) in catalysis?

**Scheme 1:**
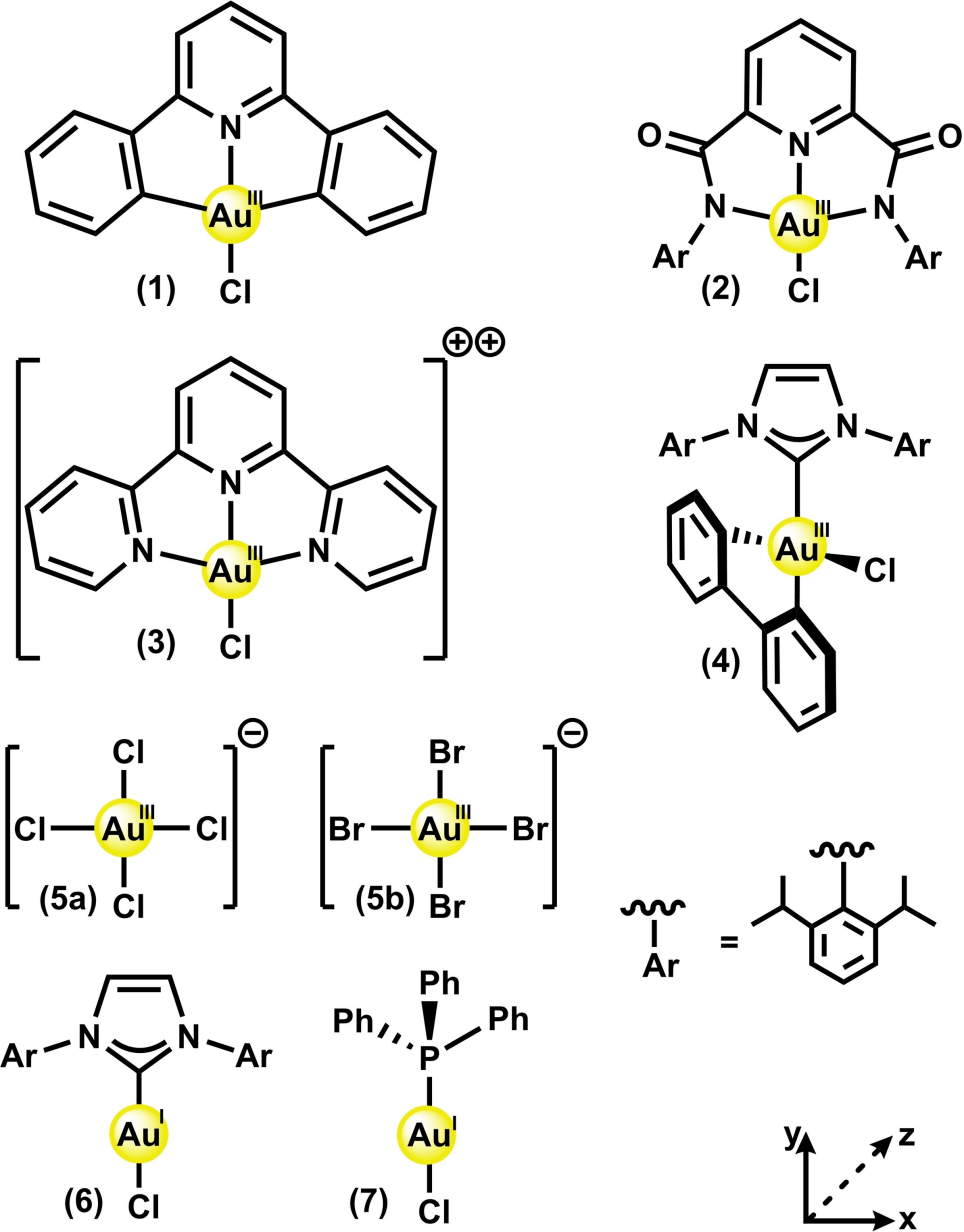
The set of formal Au^I^ complexes (**6**–**7**), Au^III^ complexes (**1**–**4**) and Au^III^ salts (**5 a**–**5 b**) investigated in this work.

In this work we investigate the electronic structure of a series of formal gold(I) and gold(III) complexes (Scheme 1), addressing (a) by applying the same computational methodology we used for copper(III)^[11]^ and (b) via Au L_3_‐edge X‐ray Absorption Near Edge Spectroscopy (XANES). Au L_3_‐edge XANES has been previously applied to study gold clusters^[14]^ and complexes,^[15]^ as it directly probes the unoccupied 5d states—providing valuable insight into the metal's d‐electron configuration. Finally, we discuss (c) in the context of future developments in the field of gold(III) catalysis.

## Results and Discussion

**Figure 1:**
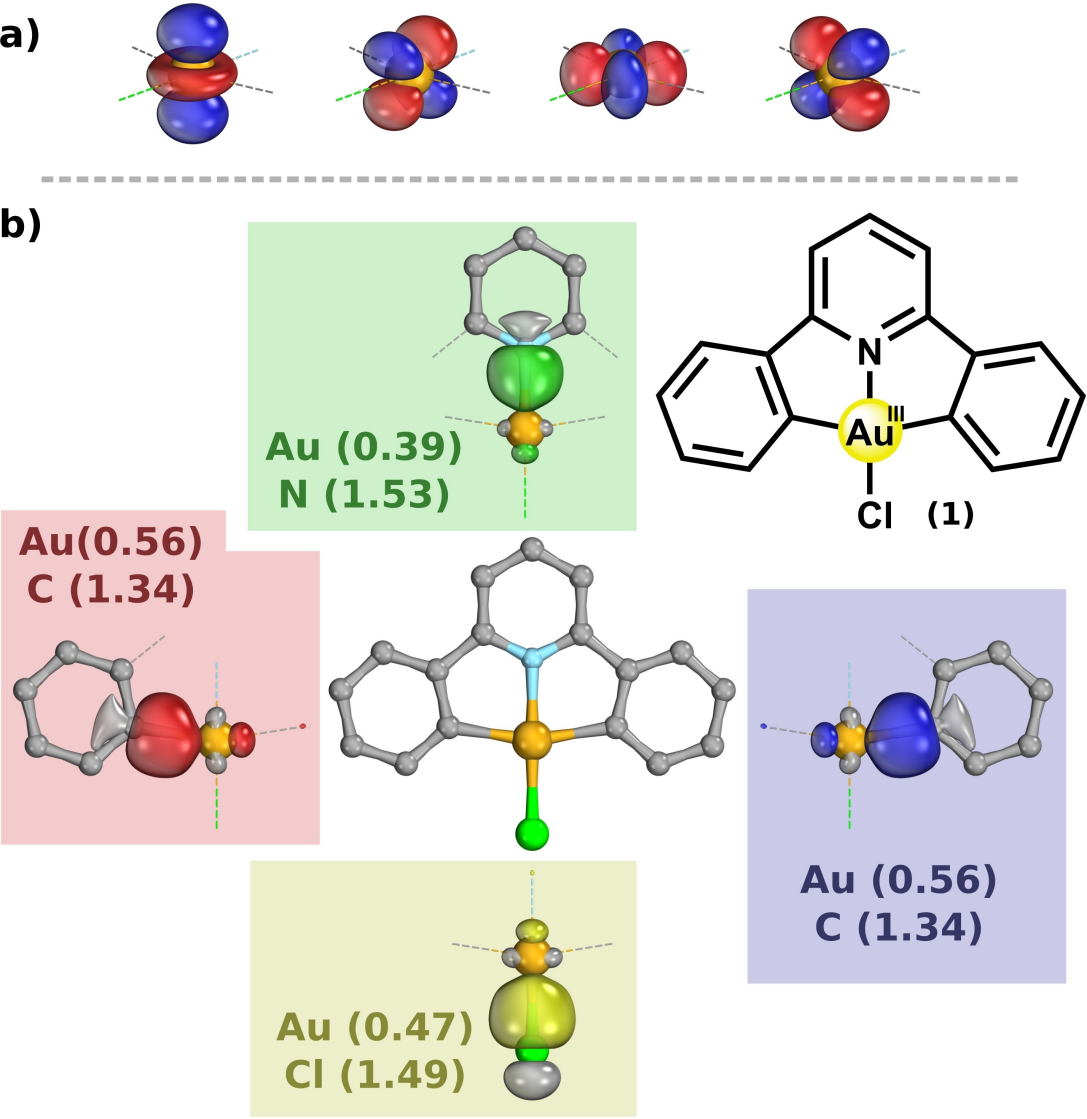
The structure of the formal gold(III) complex **(1)**, a) its intrinsic d^8^ configuration (4× δ‐IBO^2^), and b) the four doubly occupied localized metal‐ligand σ‐bonding orbitals (4× σ‐IBO^2^) with the corresponding IBO partial charge distributions. Calculated with PBE0/def2‐TZVPP//B97‐3c and rendered in IboView with isosurfaces enclosing 80 % of each orbital's electron density.

*The Intrinsic Electronic Structure of Gold(III), Identifying a Quasi‐d* ^*10*^ *Configuration*: We may gain immediate insight into the electronic structure of a typical gold(III) complex via examination of the Intrinsic Bonding Orbitals (IBOs)^[16]^ of the C^N^CAu^III^Cl complex **(1)** (Figure 1). The IBOs are localized orbitals that exactly represent a calculated wavefunction in terms of chemically convenient bonding and non‐bonding contributions. We firstly note the presence of four doubly occupied IBOs of δ‐symmetry (4× δ‐IBO^2^), well localized onto the gold center (q(Au)_δ‐IBO_ >1.96), i.e., we can identify an intrinsic d^*8*^ configuration (Figure 1a), reflecting the formal gold(III) assignment. Next, we should consider the metal‐ligand σ‐bonding framework, expressed by the four doubly occupied IBOs of σ‐symmetry (4× σ‐IBO^2^; shown in green, red, blue, and yellow in Figure 1b). Just as Snyder pointed out in the formal copper(III) case,[^8a^, ^8c^] these bonds gain non‐negligible electron‐sharing covalency by forming an admixture with the formally unoccupied d_x2‐y2_ orbital of the metal. This is consistent with the identification of a σ‐antibonding virtual valence (vv)IBO with Au 5d_x2‐y2_ character (see Table S1). We can judge the degree of electron sharing in the metal‐ligand σ‐bonding framework via inspection of the IBO partial charge distributions. For an ideal electron‐sharing bond (a non‐polar bond), we would expect one electron's worth of density ascribable to the metal, i.e., an IBO with metal partial charge M(1.00). The IBO of an ideal dative bond (polarized towards the ligand) would have a metal charge approaching zero, M(0.00). Where do the σ‐bonds of the formal gold(III) complex **(1)** lie on this bonding spectrum? Gold's partial charge in each of the σ‐IBOs, q(Au)_σ‐IBO_, lies around 0.5 (0.39–0.56), i.e., approximately halfway between the idealized dative and electron‐sharing scenarios. The total amount of electron density gold recovers through σ‐bonding, obtained by summing over these four bonds (Σq(Au)_σ‐IBO_) is 1.97e. These two electrons worth of density are in addition to the intrinsic d^*8*^ configuration, and we may thus describe this bonding scenario as a quasi‐d^10^ configuration at the metal center. This is isoelectronic to the formal copper(III) complex [Cu(CF_3_)_4_]^1−^,^[11]^ a bonding scenario that may also be expected for [Au(CF_3_)_4_]^1−^ and other (trifluoromethylated) gold(III) complexes.^[17]^

For the other formal gold(III) compounds **(1**–**5)** e.g. the individual metal IBO partial charges, q(Au)_σ‐IBO_, do change somewhat, but their sums, Σq(Au)_σ‐IBO_, and total d‐electron counts do so to a much lesser degree, indicating a similar cumulative amount of electron‐sharing in their σ‐bonding frameworks (Table 1). For example, the nature of the Au−C_phenyl_ bond in the C^N^C pincer ligand in **(1)** is slightly more electron‐sharing, as compared to the Au−N_pyridine_ bond its N^N^N analogue **(3)**. This effect is compensated by an increase in the dative character of the Au−Cl bond in **(1)**.

**Table 1:** IAO partial charge distributions of the M−L σ‐IBOs in complexes (**1**–**7**) and their sums, Σq_σ‐IBO_(M), where M=Au or Cu. Calculated with PBE0/def2‐TZVPP//B97‐3c.

Complex	q_σ‐IBO_				Σq_σ‐IBO_(M)
	Au, L_1_ (L_1_=)	Au, L_2_ (L_2_=)	Au, L_3_ (L_3_=)	Au, L_4_ (L_4_=)	
**(1)**	0.38, 1.53 (N_pyridine_)	0.47, 1.49 (Cl)	2×[0.56, 1.34 (C_phenyl_)]		1.97
**(2)**	0.39, 1.53 (N_pyridine_)	0.49, 1.47 (Cl)	2×[0.45, 1.45 (N_amide_)]		1.78
**(3)** ^[a]^	0.37, 1.52 (N_trans_)	0.60, 1.37 (Cl)	2×[0.42, 1.51 (N_cis_)]		1.81
**(4)** ^[a]^	0.25, 1.61 (C_carbene_)	0.77, 1.16 (C_cis_)	0.89, 1.07 (C_trans_)	0.18, 1.67 (Cl)	2.09
**(5 a)**	4×[0.50, 1.45 (Cl)]				2.00
**(5 b)**	4×[0.53, 1.40 (Br)]				2.12
**(6)**	0.44, 1.52 (C_carbene_)	0.33, 1.64 (Cl)	n/a		0.77
**(7)**	0.45, 1.50 (P)	0.34, 1.62 (Cl)	n/a		0.79
**[Cu(CF_3_)_4_]^1− [b]^**	4×[0.46 (Cu), 1.47 (C)]				1.84

[a] *trans/cis* with respect to the chloride (see Scheme 1). [b] values from ref. [11] (calculated with the same level of theory).

Like IBO analysis, Energy Decomposition Analysis (EDA)^[18]^ is an effective tool to understand computed wavefunctions, although it does so energetically—rather than spatially. At the optimized geometry, a molecule is spilt into user‐defined fragments. As Hopffgarten and Frenking explain,^[19]^ the instantaneous (electronic) interaction energy (Δ*E*_int_) is simply the difference between the energies of the molecule and the fragments (eq. 1). The essence of EDA is that Δ*E*_int_ is split up, or decomposed, into chemically interpretable contributions: the quasiclassical electrostatic interaction energy, Δ*E*_elstat_; the Pauli exchange repulsion, Δ*E*_Pauli_; and the orbital mixing term, Δ*E*_orb_ (eq. 2). The orbital mixing term (also referred to as the orbital interaction energy) is the most relevant to the present purpose as it measures how much the orbitals of the fragments change (mix) upon molecular formation and can therefore be used to judge the suitability of a chosen fragmentation scheme. A smaller Δ*E*_orb_ means less energetic gain due to orbital mixing, i.e., the chosen fragment states are more similar to the relaxed molecule.

**Figure 2:**
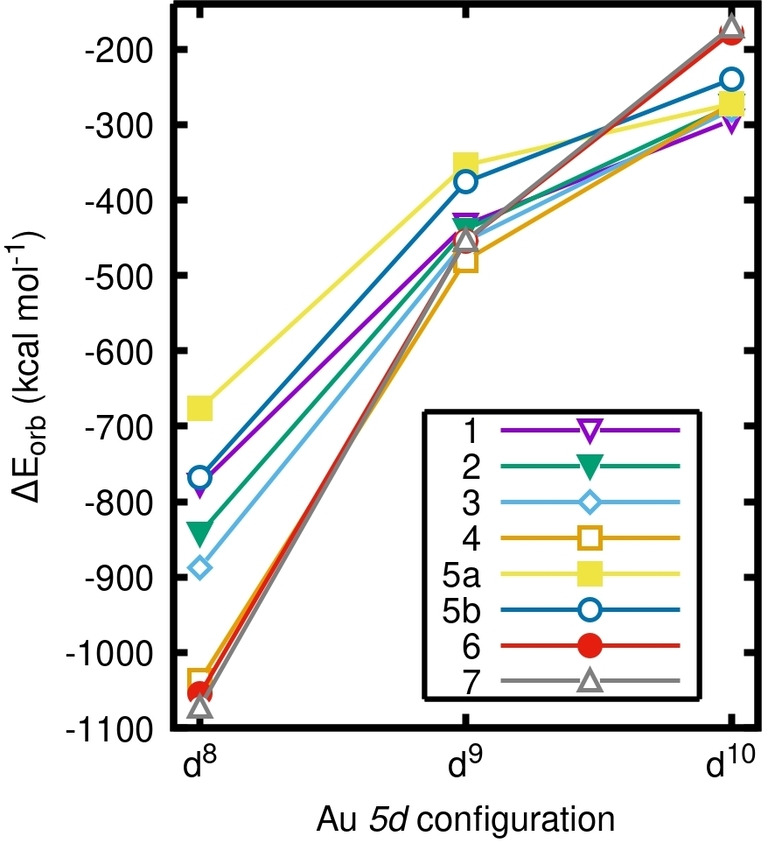
The orbital interaction energies (Δ*E*_orb_) vs. the gold configuration for the set of compounds **(1**–**7)**, showing that 5d^10^ is most favorable in all cases. Calculated with PBE0‐ZORA/TZ2P//B97‐3c.

To perform comparative EDA, we chose to fragment the compounds **(1**–**7)** into two fragments: the metal (Au^*n+*^) and the ligand(s) (L^*n−*^). The electronic configuration of the metal fragment was varied from d^8^ to d^10^ by changing its charge (*n*=1, 2, 3) and enforcing local symmetry (see Supporting Information for further details). Splitting the formal gold(III) complex **(1)** into (5d^10^ Au^**+**^ & L^**−**^) fragments is found to be most favorable, as its Δ*E*_orb_ is smaller than both the (5d^9^ Au^**2+**^ & L^**2−**^) and (5d^8^Au^**3+**^ & L^**3−**^) fragmentations by several hundred kcal mol^−1^ (Figure 2, purple). In fact, the EDA of the formal gold(III) complexes **(1**–**5)** reveals that fragmenting to a 5d^10^ metal center is most favorable in every case (Figure 2). We can therefore confirm the identification of a quasi‐d^10^ configuration for all the investigated formal gold(III) compounds **(1**–**5)**. This finding is contrasted by e.g., nucleophilic gold complexes, which have effective 5d^10^6s^1^ configurations, as identified by comparative EDA at the same level of theory.^[16g]^

**Figure 3:**
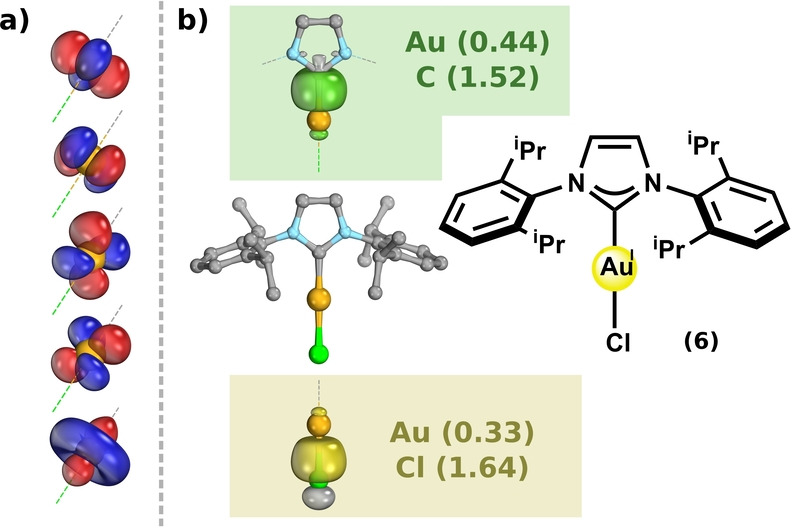
IBO analysis of a typical formal gold(I) complex **(6)**. The intrinsic d^10^ configuration (5× δ‐IBO^2^) (a), two doubly occupied localized metal‐ligand σ‐bonding orbitals (2× σ‐IBO^2^) and the corresponding IBO partial charge distributions (b). Calculated with PBE0/def2‐TZVPP//B97‐3c and rendered in IboView with isosurfaces enclosing 80 % of each orbital's electron density.

*Classical d* ^*10*^ *Gold(I)*: The IBOs of a typical gold(I) complex, the *N*‐heterocyclic carbene (NHC) gold(I) chloride complex NHC−Au−Cl **(6)** (Figure 3), reveal a markedly different bonding scenario to that seen for gold(III). We can immediately identify an intrinsic d^10^ configuration (Figure 3a), supporting the formal ***OS*** assignment. In addition, there are two metal‐ligand σ‐bonds (Figure 3b), which have non‐negligible contributions from the gold center. Unlike in the gold(III) case however, these cannot involve participation of the gold 5d_x2‐y2_ orbital, as the entire d‐manifold is fully occupied. Rather, the gold 6s orbital is implicated, consistent with the considerable Au 6s character visible in the σ‐antibonding vvIBO (see Supporting Information for further details). The Δ*E*_orb_ from the EDA of the formal gold(I) complexes **(6)** and **(7)** (Table 2) shows that fragmenting into the 5d^10^6s^0^ state is most favorable, here matching both the intrinsic d‐configuration and the formal ***OS***. The participation of the 6s in the σ‐bonding of gold(I), seen in the IBO analysis, can be further probed by including [Xe]4f^14^5d^10^6s^1^ fragment states in the EDA. Notably, the Δ*E*_orb_ of the [Xe]4f^14^5d^10^6s^1^ fragment states of **(6)** and **(7)** (−298 and −258 kcal mol^−1^, respectively) indicates they are in relatively close competition with the [Xe]4f^14^5d^10^6s^0^ configuration (−178 and −170 kcal mol^−1^, respectively).^[20]^ This is expected, from the understood effects of lanthanide and relativistic contraction, resulting in poor energetic separation of the valence s and d shells in gold,^[21]^ as compared to the other coinage metals. Indeed, IBO analysis (Figure S2) and EDA (Table S5) of the homologous analogues of the NHC−Au−Cl **(6)** (NHC−Ag−Cl and NHC−Cu−Cl), indicate diminished participation of the valence s*‐*orbital in the metal‐ligand σ‐bonding.

**Table 2:** Orbital interaction energies (Δ*E*_orb_), in kcal mol^−1^, per gold fragment state^[a]^ from the EDA, calculated with PBE0‐ZORA/TZ2P//B97‐3c.

Complex	d^8^s^0^	d^9^s^0^	d^10^s^0^	d^10^s^1^
**(6)**	−1054.96	−454.06	−178.02	−297.50
**(7)**	−1072.11	−453.79	−170.22	−258.54

[a] Abbreviated electronic configuration e.g., d^8^s^0^ *=*[Xe]4f^14^5d^8^6s^0^.

*Spectroscopic Signatures of d* ^*10*^ *Gold(I) vs. Quasi‐d* ^*10*^ *Gold(III)*: Now that the quasi‐d^10^ description for gold(III) complexes has been established based on our computational analysis above, we may ask whether we can observe excitation into the d‐orbital manifold. To address this question, we applied Au L_3_‐edge X‐ray Absorption Near Edge Spectroscopy (XANES). The so‐called whiteline absorption feature of a Au L_3_‐edge XANES spectrum (ca. 11 915–11 924 eV) formally corresponds to the 2p→5d transition.^[22]^ Since the metal‐centered 2p orbitals of gold are fully occupied, the strength of this absorption feature correlates with the density of the unoccupied 5d states i.e., it is not observed for metallic gold with its d‐orbitals fully occupied (5d^10^). In the case of molecular compounds, such as **(1)**–**(7)**, these final states can either be fully localized on the metal or hybridized in covalent bonds with the ligands, depending on the nature of the metal‐ligand bonding.

**Figure 4:**
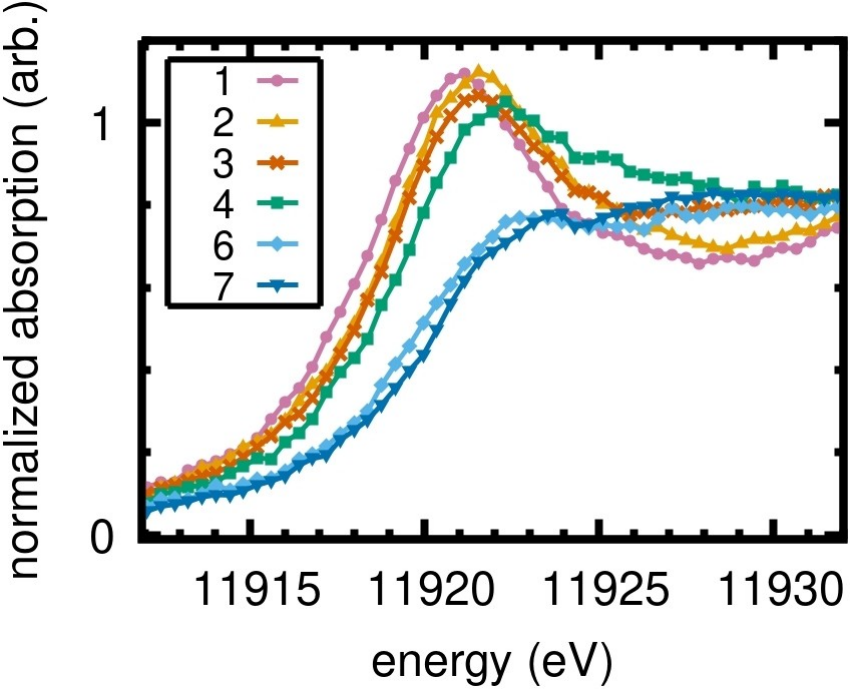
XANES spectra of the formal gold(I) complexes **(6)**–**(7)** and formal gold(III) complexes **(1)**–**(4)**.

The XANES spectra of the two gold(I) complexes **(6)** and **(7)** (light and dark blue in Figure 4, respectively) show an edge step with essentially no whiteline absorption feature. By contrast, the gold(III) complexes **(1)**–**(4)** all show similarly strong whiteline absorptions. The L_3_‐edge peak heights of the tetrahaloaurate salts **(5 a)** and **(5 b)** are somewhat anomalous as they are significantly lower than those of the gold(III) complexes, although the whiteline absorption features are still clearly visible (see Supporting Information for further details). The formal gold(III) complexes **(1)**–**(4)** exhibit remarkably similar absorption patterns, further demonstrating a unity in their electronic structures. The formal gold(I) complexes **(6)** and **(7)** are equally similar to each other. While no simple relationship was found between the edge position and the metal's ***OS*** (Table S1), the spectroscopic fingerprints of gold(I) and gold(III) complexes are clearly distinct. Specifically, strong whiteline absorptions are seen for all the gold(III) complexes, due to metal‐localized final 5d^10^ states. This reflects the incomplete occupation of the 5d‐orbitals in their (initial) ground state, clearly demonstrating that d^10^ is not an adequate description for gold(III) complexes. For the gold(I) complexes, with their initial 5d^10^ states, no such excitation is possible and thus no whiteline is observed. This interpretation of Au L_3_‐edge absorption data is consistent with previously reported high‐energy‐resolution fluorescence‐detected XAS of Au/Al_2_O_3_,^[23]^ which additionally probed participation of the 6s orbital.

The computational data (see above) showed that the gold(III) centers in **(1)**–**(4)** all have intrinsic d^8^ configurations. Despite this, they most easily fragmented into Au d^10^ centers in the EDA, due to the electron‐sharing covalent contributions to the metal‐ligand σ‐bonds, i.e., quasi‐d^10^ configurations were identified. From the pronounced whiteline absorption features in Au XANES data of these gold(III) complexes, we can conclude that excitation into the valence d‐space is indeed still possible, further justifying the distinction between quasi‐ and classical‐d^10^ configurations. Although the number of complexes studied here is modest, we believe the chosen complexes are representative of typical gold(I) and gold(III) systems. For example, replacing the amide moieties in **(2)** with carboxylate groups in (pyridine‐2,6‐dicarboxylato)Au^III^Cl leads to a very similar picture (Figure S3, Table S6), albeit one with slightly more classical (dative) metal‐ligand bonding, indicating that our findings can equally be transferred to complexes featuring oxygen‐based ligands. While these results clearly demonstrate the unsuitability of the classical‐d^10^ description for the formal gold(III) complexes studied here, the quasi‐d^10^ label is still recommended to indicate the heighted electron‐sharing nature of their metal‐ligand bonding. This is contrasted by gold complexes with more classical dative bonds e.g., fluorido gold complexes,^[24]^ which are much more reactive than typical gold(III) complexes, presumably due to their increased d^8^ character.

*The Role of Gold(I) vs. Gold(III) in Catalysis*: In 2004, Straub reflected on the fact that both gold(I) and gold(III) had been suggested as the active species in several gold‐catalyzed reactions.^[25]^ DFT calculations showed that the barriers of cyclization and cycloaddition reactions promoted by gold(I) and gold(III) are very similar, leaving open the question of the active species’ ***OS***. Jones et al. came to similar conclusions that same year, when examining C−H bond activation reactions.^[26]^ The lack of distinction between the reactivity of gold(I) and gold(III) manifests experimentally in a wide range of reactions.^[2]^ Some representative examples are the catalytic transformations of bicyclo[3.2.1]octene derivatives,^[27]^ the cyclization reactions of *N*‐propargyl tryptophans or tryptamines,^[28]^ and the auration of electron rich (hetero)arenes.^[29]^ Since the ***OS*** formalism is supposed to summarize chemical behavior, the similarity of gold(I) and gold(III) catalysis is quite remarkable and may lead to differing explanations. For example, gold‐catalyzed reactions, where gold acts electrophilically as a Lewis acid (as distinct from stoichiometric or catalytic transformations where the ***OS*** of gold changes e.g., gold(I)/gold(III) couples),[^5^, ^6^, ^30^] it has been proposed that gold(III) is a precatalyst, transforming into the active gold(I) species through in situ reduction.^[31]^ While it was a prominent idea in the earlier days of the field, this hypothesis has not been further discussed much, until being recently questioned.^[32]^ Notably too, high‐valent complexes have previously been shown to be competent catalysts in their own right, retaining their +III oxidation state in situ.^[15a]^ The computational and spectroscopic analysis presented here should dismiss the in situ reduction suggestion, as clear differences between the electronic structures of gold(I) and gold(III) centers were found both computationally and spectroscopically. Even in the Energy Decomposition Analysis, where we found that both gold(I) and gold(III) most easily fragment to a d^10^ configuration, the average ease of doing so (measured by the orbital interaction energies, Δ*E*_orb_) varied greatly (−174 vs. −272 kcal mol^−1^, respectively).

The question remains: Why do we sometimes see such similar chemical behavior from gold in different ***OS*** s? The Lewis acidic properties of gold(I), responsible for its renowned ability to activate a variety of substrates, derives from the formally unoccupied 6s orbital's participation in bonding. The 6s orbital of gold(I) is known to be particularly energetically accessible due to relativistic effects and lanthanide contraction.^[21]^ Conversely, the ability of gold(III) to strongly activate substrates comes from its desire to adopt a d^10^ configuration. This desire is left unfulfilled, resulting in a quasi‐d^10^ configuration and a more reduced metal center. These strikingly different causes have the same outcome: increased electron‐sharing covalency in the metal‐ligand σ‐bonding framework. This shared mechanism activates substrates, making them more electron‐poor, i.e., vulnerable to (nucleophilic) attack. Thus, gold(I) and gold(III) centers may exhibit very similar reactivity for very different reasons. With this understanding in hand, we can appreciate how the +III OS of gold can constitute an active catalyst, where the *a priori* assumption that a gold(I) species is formed via in situ reduction may be misleading. These conclusions echo those recently drawn by Lopez and co‐workers.^[32]^

Much initial work in enantioselective gold catalysis focused on the use of chiral gold(I) complexes, with few notable gold(III) systems.^[33]^ More recently, there has been an upswing in the use of gold(III) complexes,^[34]^ which are in general better suited to this purpose thanks to their more sterically encumbered square planar geometries. The bonding scenarios uncovered herein explain the origin of the transferable reactivity patterns available to gold in these oxidation states, a finding that holds promise for this flourishing field.

While the generalization that gold(I) and gold(III) may be used interchangeably as catalysts broadly holds, we do note that in certain cases divergence in the chemical behavior can be observed e.g., the oxidation chemistry of gold hydroxide complexes.^[35]^

## Conclusion

Through analysis of computational and spectroscopic data, the electronic structures of several representative gold(I) and gold(III) complexes are probed. Our results support classical d^10^ and quasi‐d^10^ descriptions of formal gold(I) and gold(III) centers, respectively. We detailed the computational and spectroscopic distinctions between quasi‐d^10^ and classical d^10^ configurations. The quasi‐d^10^ label communicates the large cumulative degree of electron‐sharing covalency in the σ‐bonding framework, while acknowledging that a genuine d^10^ configuration is not reached (contrasting the implications of the inverted ligand field description). The quasi‐d^10^ bonding motif seems to be common in complexes with coinage metals in the +III formal ***OS***, e.g., [Cu(CF_3_)_4_]^1−^.^[11]^ Non‐negligible electron‐sharing nature was also seen in the σ‐bonding framework of formal gold(I) complexes examined, originating from the low‐lying 6s orbital. Thus, despite their physically distinct electronic structures, we can now understand why gold(I) and gold(III) complexes tend to activate substrates to similar extents, and therefore often exhibit very similar reactivities.

## Conflict of interest

The authors declare no conflict of interest.

1

## Supporting information

As a service to our authors and readers, this journal provides supporting information supplied by the authors. Such materials are peer reviewed and may be re‐organized for online delivery, but are not copy‐edited or typeset. Technical support issues arising from supporting information (other than missing files) should be addressed to the authors.

Supporting Information

Supporting Information

## Data Availability

The data that support the findings of this study are available in the supplementary material of this article.
