# Supplementary material for: Spectroscopic Manifestations and Implications for Catalysis of Quasi‐d10 Configurations in Formal Gold(III) Complexes
Source: Angew Chem Int Ed Engl. 2022 Dec 12;62(3):e202215523. doi: 10.1002/anie.202215523 (PMC10107628; doi:10.1002/anie.202215523)
Supplement: Supplementary file 1 — Supporting Information [file ANIE-62-0-s002.pdf]

Angewandte Chemie
Eine Zeitschrift der Gesellschaft Deutscher Chemiker

Supporting Information

Spectroscopic Manifestations and Implications for Catalysis of Quasi-d^{10} Configurations in Formal Gold(III) Complexes

E. A. Trifonova, I. F. Leach, W. B. de Haas, R. W. A. Havenith, M. Tromp, J. E. M. N. Klein**

Table of Contents

**1. Au L_3-edge XAS measurements**	S2
**2. Computational details**	S3
**General**	S3
**Example Inputs**	S4
**Energy Decomposition Analysis**	S4
**Choice of Orbitals and vv-IBOs**	S5
**Comparison of NHC-M-Cl (M = Cu, Ag, Au)**	S8
**3. Synthesis and characterization of compounds (1)-(8)**	S8
**4. Optimized Cartesian coordinates of compounds (1)-(8)**	S9
**5. Oxygen-based ligands**	S15
**References**	S16

1. Au L_3-edge XAS measurements

XAFS measurements of the samples were performed using a commercially available X-ray spectrometer EasyXES100 by EasyXAFS LLC, which was adjusted to our needs. The spectrometer was used in an absorption mode. As the source, a Varian VF-50J X-ray tube was used with a Pd-target that was powered by a high voltage Spellman supplier (ux Series) at 25 kV and 2 mA. As the detector, a Vortex-60 EX detector by Hitachi was used. To adjust the width of X-ray beam going to SBCA and then to the detector, 3mm slits were installed just after the exit window of the X-ray tube and in front of the entrance window of the detector. To reduce scattering from air, a metal tank filled with He gas, that had some windows covered with Kapton film, was placed in the center of the Rowland circle geometry.[1] The whole X-ray spectrometer set-up was shielded with a 3 mm steel enclosure for safety. We used a spherically bent Si 773 crystal of Johan's type[2] produced by XRS TECH LLC. This allowed us in principle to obtain absorption spectra in the energy range of 11 860 to 13 000 eV. For our samples, two series of Au L_3-edge XAS measurements were performed. First, the measurements were done in an energy range of 11860-13000 eV with a step of 1.0 eV and the measuring time for each step of 1.0 s. Then, the energy range was chosen to be 11860 to 12300 eV with a step of 0.4 eV and the measuring time for each step of 1.0 s. To prepare complexes (**1**)-(**7**) for measurement, each sample was ground for ~10 minutes with a pestle and mortar, mixed with 50-60 mg of boron nitride and pelleted into a 2 mm sample holder, having a window of 10 mm in diameter. Differences in absorbance between samples was minimized by adjusting the weight of each compound such that it corresponded to 5 mg of gold. The XAS measurements were carried out under ambient conditions (~298 K, ~1 atm). NMR measurements before and after confirmed a lack of sample decomposition. All spectra were calibrated with respect to Au foil. For each sample, 12 scans were measured that were then averaged to reduce statistical errors. For the I0 measurements, 30 scans were carried out and then averaged.

The XAS data was processed and analyzed with the Demeter package.[3] In Athena, the pre-edge was defined as -55 to -30 eV and the normalization (post-edge) range was set from +50 to +300 eV (with respect to E_0). A normalization order of 2 (linear) was used, with a k-weight of 2. No low-end spline clamps were used, while high-end spline clamps were set to *Strong*. Normalization was verified by visually examining the extended XAS region, which was found to approach unity for all samples (Figure S1).

Table S1: Au L_3-edge energies (in eV), as determined by the maximum in the 1st derivative of the absorption with respect to energy.

Complex	Edge position
1	11919.(3)
2	11919.(0)
3	11918.(8)
4	11919.(7)
5a	11918.(3)
5b	11919.(1)
6	11919.(0)
7	11920.(4)

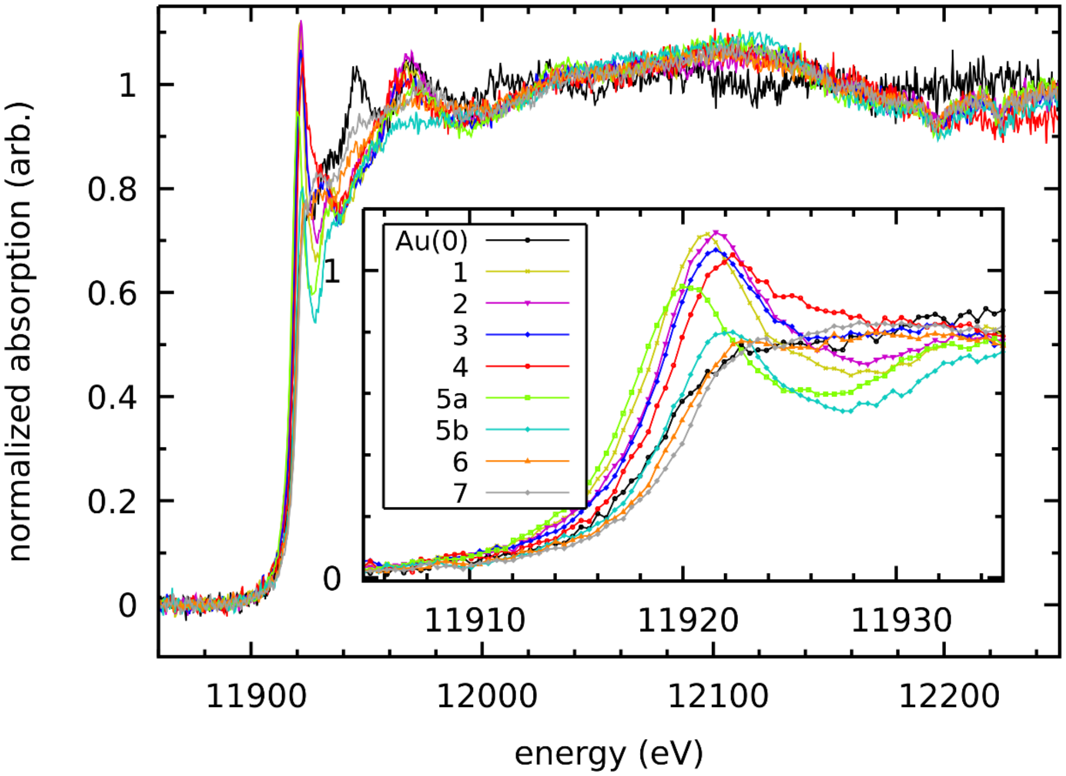

Figure S1: The normalized Au L_3-edge XAS spectra of all species **(1)-(7)** and gold foil, Au(0). The inset shows the edge region.

2. Computational details

General

Calculations were performed in ORCA 4.2.1.[4] Geometries were optimized with the efficient composite B97-3c method from Grimme,[5] which is based Becke's 1997 local exchange correlation functional[6] and uses the triple-zeta mTZVP basis set (based on the Ahlrichs basis set def2-TZVP)[7] and the corresponding effective core potential,[8] to model relativistic effects by replacing the inner 60 electrons of Au. (Geometric) dispersion effects are accounted for via the D3 model.[9] Analytical frequencies were computed, and all structures were found to be well defined minima i.e. all calculated frequencies were positive. An energetic convergence criterion of 10^{-8} au was request via the *TightSCF* keyword. An increased accuracy of the DFT integration grid was requested with the *Grid5 NoFinalGrid* keywords.

Intrinsic bonding basis analysis (IBBA)[10] and was performed in IboView,[11] via additional single point calculations which were performed at the (B97-3c) optimized geometries with the PBE0[12]/def2-TZVPP[7] level of theory. The *RIJCOSX* approximation[13] was employed to speed up the integral evaluation, using Weigend's universal fitting basis set (*def2/J*). An energetic convergence criterion of 10^{-8} au was requested (*TightSCF*). An increased accuracy of the DFT integration grid was requested (*Grid5 NoFinalGrid*). Structural depictions and orbital visualization of all species were produced with IboView[11] (source code available at http://www.iboview.org/). Orbital were localized with *exp 2*.

Examples inputs

**Geometry optimization**	*! RKS B97-3c Opt Freq Grid5 NoFinalGrid TightSCF*
**Single point calculations**	*! PBE0 def2-TZVPP def2/J RIJCOSX Grid5 NoFinalGrid TightSCF NormalPrint*

Energy Decomposition Analysis

To further probe the electronic structure, and in particular the Au *d*-configuration, Morokuma-Ziegler Energy Decomposition Analysis (EDA)[14] was performed in the Amsterdam Density Functional (ADF) suite of the AMS 2020 package.[15] These calculations were performed at the B97-3c optimized *n=1* geometry from ORCA, and employed the PBE0 functional[12] in combination with the triple-ζ TZ2P basis set.[16] No frozen core approximation was made. Scalar relativistic effects were modelled *via* a ZORA Hamiltonian.[17] Numerical quality was defined with the *Good* keyword. For each EDA calculation only two fragments were defined: 1) the metal centre, Au^{n+} and 2) the entire remaining ligand framework $L^{(n+1)-}$. The optimised coordinates were reoriented such that the Au-L bonds lay along the *xy* axes, providing optimal overlap between the ligand and metal ($3d_{x2-y2}$) orbitals. Although no symmetry was enforced during the complex and ligand calculations, local D_{4h} symmetry was applied to the metal fragment to enable specification of the $6s^05d^8$, $6s^05d^9$ and $6s^15d^{10}$ electronic configurations *via* the *IrrepOccupations* keyword.

Table S2: EDA of all species gold **(1)**-**(8)** (plotted in Figure 2) and **NHC-M-Cl** (M = Ag, Cu), calculated with PBE0-ZORA/TZVP//B97-3c. Along with the instantaneous interaction energy (ΔE_{int}) the orbital interaction energy (ΔE_{orb}), the quasiclassical Coulomb interaction (ΔE_{elstat}) and repulsive Pauli exchange (ΔE_{Pauli}) terms are also given. All energies are in kcal mol^{-1}. For each species, the most favourable configuration (with the smallest ΔE_{orb}) is emphasized in bold.

Species	Au configuration	ΔE_{int}	ΔE_{orb}	ΔE_{elstat}	ΔE_{Pauli}
**(1)**	[Xe]$4f^{14}5d^86s^0$	-1197.43	-777.1	-763.65	343.33
	[Xe]$4f^{14}5d^96s^0$	-449.22	-432.58	-377.02	360.39
	[Xe]$4f^{14}5d^{10}6s^0$	-172.13	**-294.2**	-264.23	386.29
**(2)**	[Xe]$4f^{14}5d^86s^0$	-1644.41	-842.01	-1128.28	325.88
	[Xe]$4f^{14}5d^96s^0$	-764.94	-439.18	-668.14	342.37
	[Xe]$4f^{14}5d^{10}6s^0$	-309.34	**-275.15**	-426.35	392.15
**(3)**	[Xe]$4f^{14}5d^86s^0$	-1815.97	-887.55	-1323.88	395.45
	[Xe]$4f^{14}5d^96s^0$	-866.89	-454.32	-782.64	370.07
	[Xe]$4f^{14}5d^{10}6s^0$	-363.57	**-279.94**	-475.71	392.08
**(4)**	[Xe]$4f^{14}5d^86s^0$	-1825.64	-1037.33	-1192.84	404.54
	[Xe]$4f^{14}5d^96s^0$	-877.59	-479.6	-795.62	397.63
	[Xe]$4f^{14}5d^{10}6s^0$	-372.96	**-272.18**	-511.32	410.53
**(5a)**	[Xe]$4f^{14}5d^86s^0$	-2010.56	-676.26	-1586.38	252.09
	[Xe]$4f^{14}5d^96s^0$	-981.83	-353.63	-896.03	267.83
	[Xe]$4f^{14}5d^{10}6s^0$	-412.29	**-272.48**	-457.15	317.35
**(5b)**	[Xe]$4f^{14}5d^86s^0$	-1946.23	-767.71	-1412.64	234.13
	[Xe]$4f^{14}5d^96s^0$	-941.22	-376.16	-804.64	239.58
	[Xe]$4f^{14}5d^{10}6s^0$	-380.76	**-240.03**	-417.53	276.79
**(6)**	[Xe]$4f^{14}5d^86s^0$	-1771.15	-1054.96	-1000.96	284.77
	[Xe]$4f^{14}5d^96s^0$	-833.66	-454.06	-671.48	291.87
	[Xe]$4f^{14}5d^{10}6s^0$	-283.04	**-176.44**	-413.99	307.39
	[Xe]$4f^{14}5d^{10}6s^1$	-139.8	-297.5	-307.99	465.7
**(7)**	[Xe]$4f^{14}5d^86s^0$	-1758.86	-1072.11	-944.03	257.28
	[Xe]$4f^{14}5d^96s^0$	-806.74	-453.79	-612.41	259.45
	[Xe]$4f^{14}5d^{10}6s^0$	-266.04	**-170.22**	-368.99	273.17
	[Xe]$4f^{14}5d^{10}6s^1$	-126.1	-258.54	-286.27	418.71

**(8)**	[Xe]$4f^{14}5d^{8}6s^{0}$	-1673.26	-708.17	-1272.41	307.32
	[Xe]$4f^{14}5d^{9}6s^{0}$	-762.19	-381.43	-702.44	321.67
	[Xe]$4f^{14}5d^{10}6s^{0}$	-311.36	**-277.55**	-393.48	359.67
	[Xe]$4f^{14}5d^{10}6s^{1}$	-169.79	-418.50	-336.03	584.74
**NHC-Ag-Cl**	[Kr]$4d^{10}5s^{0}$	-226.66	**-109.59**	-322.24	205.18
	[Kr]$4d^{10}5s^{1}$	-120.99	-238.26	-224.42	341.69
**NHC-Cu-Cl**	[Ar]$3d^{10}4s^{0}$	-264.05	**-127.18**	-332.13	195.26
	[Ar]$3d^{10}4s^{1}$	-145.75	-279.62	-242.82	376.7

Choice of Orbitals and vv-IBOs

Any division of a many electron wavefunction into a set of three dimensional one-electron functions (orbitals) is ultimately an arbitrary choice. However, some choices are more appropriate for a given purpose. As bonds are understood on the basis of local interactions in molecules, an intuitive representation of a calculated wavefuction can be obtained via localization of the canonical orbitals. Several different localization criteria exist e.g. Foster-Boys,[18] Ruedenberg-Edmiston[19] but here we choose to calculate the intrinsic bonding orbitals (IBOs), which employ the Pipek-Mezey localization procedure[20] that maintains the σ/π symmetry distinction. Furthermore, thanks to a projection onto a minimal basis, IBOs show little basis set dependence.[10-11] Thus, localization collects the scattered bonding contributions and yields a low number of σ-bonding orbitals between the metal and ligand fragments, which can readily be interpreted. With the use of virtual valence (vv-)IBOs, the same can be done for the (σ-)antibonding contributions, as shown by Steen *et al*.[21]

Table S3: Unoccupied vv-IBOs of **1**-**7**, calculated at the PBE0/def2-TZVPP//B97-3c level. For **(1)**-**(4)**, two σ-antibonding orbitals are seen with predominant Au *6s* character (left) and Au *5d* character (right). For **(6)**-**(7)**, the only σ-antibonding vv-IBO has predominant Au *6s* character, due to the formal d^{10} configuration. For **(5)**, the analysis is complicated by the highly symmetric geometry. Under each vv-IBO, the partial orbital charge ($q_{\sigma\text{-IBO}}$) of the metal is given.

**1**	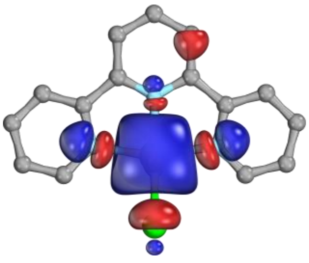<br>**Au(1.49)**	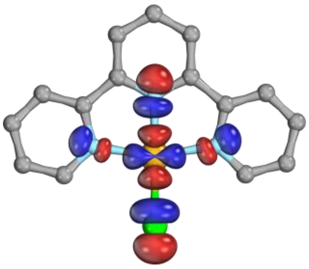<br>**Au(0.82)**
**2**	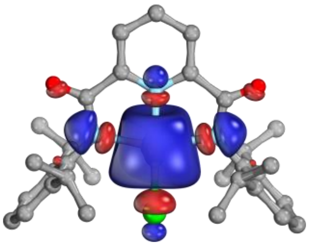<br>**Au(1.57)**	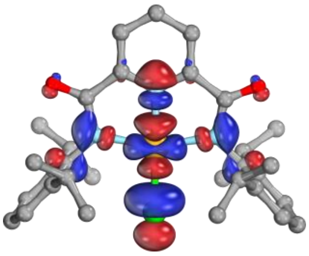<br>**Au(0.81)**
**3**	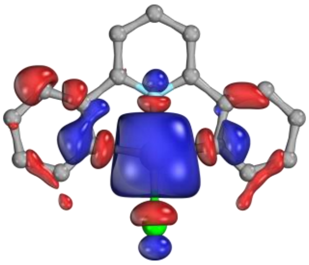<br>**Au(1.59)**	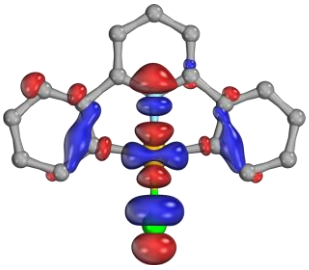<br>**Au(0.80)**
**4**	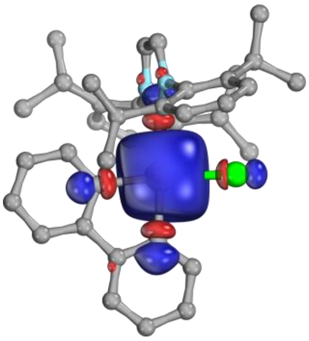<br>**Au(1.83)**	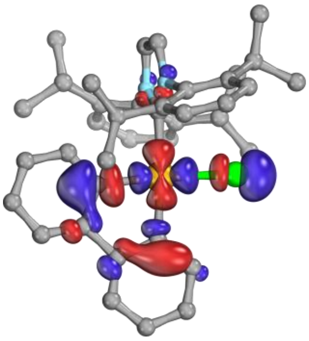<br>**Au(0.70)**
**5a**	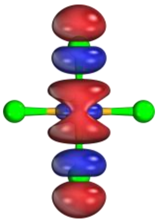<br>**Au(1.00)**	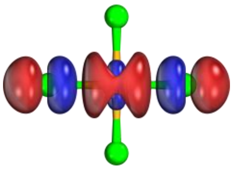<br>**Au(1.00)**

**5b**	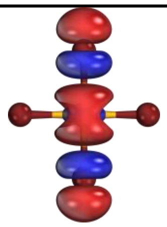 **Au(0.92)**	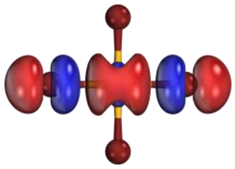 **Au(0.92)**
**6**	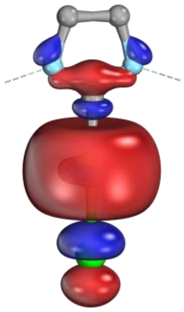 **Au(1.63)**	
**7**	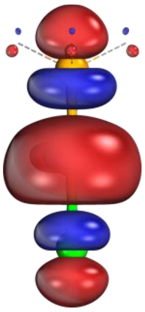 **Au(1.19)**	

Table S4: IAO partial charges and electron counts, calculated with PBE0/def2-TZVPP//B97-3c.

**Structure**	**Au partial charge**	**IAO Au electron counts**		
		**s**	**p**	**d**
**1**	1.06	2.80	6.00	9.14
**2**	1.08	2.79	6.00	9.13
**3**	1.16	2.77	6.00	9.08
**4**	0.97	2.84	6.00	9.20
**5a**	0.99	2.76	6.00	9.25
**5b**	0.85	2.79	6.00	9.36
**6**	0.36	3.06	6.00	9.58
**7**	0.29	3.02	6.00	9.68

Comparison of NHC-M-Cl (M = Cu, Ag, Au)

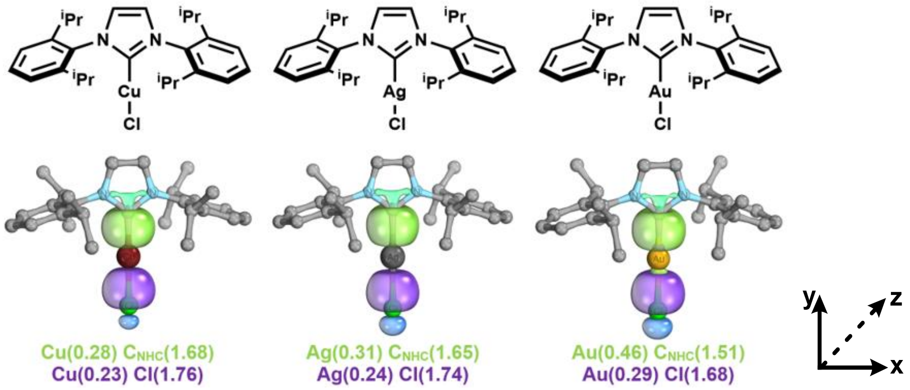

Figure S2: IBO analysis of **(6)**, compared to its silver (centre) and copper (left) analogous.

In addition to the 2 x (σ-IBO)[2] shown above in green and purple, each of the three complexes shows an intrinsic d^{10} configuration, reflecting their formal ***OS*** assignments. However, when moving down the coinage metals (towards gold), an increase in the electron-sharing nature of the metal-ligand σ-bonds is seen e.g. $q_{\sigma\text{-}IBO}(M)$ goes from 0.28 (for M = Cu) to 0.46 (for M = Au). To further probe this finding, EDA calculations were performed (see 'Computational Details' above for more details). The results (Table S5) corroborate the IBO data: the outmost valence *s*-orbital (*4s*, *5s*, and *6s*, for Cu, Ag, and Au, respectively) participates much more in bonding for Au as compared to Ag and Cu. This agrees with expectations based on lanthanide and relativistic contraction of the *6s* orbital.[22] Note, when M = Au, the complex is complex **(6)**.

Table S5: Orbital interaction energies, ΔE_{int} (in kcal mol^{-1}), of NHC-M-Cl (M = Cu, Ag, Au), from the EDA, calculated with PBE0/TZ2P//B97-3c.

M Configuration	Cu	Ag	Au (6)
…$d^{10}s^0$	-127.18	-109.59	-176.44
…$d^{10}s^1$	-279.62	-238.26	-297.50
$\Delta E_{int}(d^{10}s^0)$ - $\Delta E_{int}(d^{10}s^1)$	152.44	128.67	121.06
$\frac{\Delta E_{int}(d^{10}s^1)}{\Delta E_{int}(d^{10}s^0)}$	2.14	2.17	1.69

3. Preparation of (1)-(8)

Complexes **(1)**-**(4)** and **(6)**-**(8)** were prepared according to reported procedures.[23] The tetrahaloaurate salts **(5)** were purchased from commercial chemical suppliers as potassium salts.

The [(terpyridyl)AuCl]$(PF_6)_2$ complex (**3**) was prepared by a modification of a reported procedure.[24] Open to air $KAuCl_4$ (38 mg, 0.10 mmol, 1.0 eq.) and terpyridine (23 mg, 0.10 mmol, 1.0 eq.) were combined in H_2O (10 ml) and the pH of the resultant suspension was adjusted to 3 with HCl (1M). The reaction mixture was refluxed for 24 h. Then a solution of NH_4PF_6 (100 mg, 0.61 mmol) in 1 ml of H_2O was added and the reaction mixture was refluxed for additional 2 h. After cooling to room temperature, the yellow precipitate was collected by filtration and washed with H_2O (3×2 ml), Et_2O (2×3 ml) and dried in vacuum.

Yield 57 mg (0.075 mmol, 75%); m.p. 293.5–298.3 with decomposition.

^{1}H NMR (400 MHz, DMSO-d_6) δ 9.22 (d, J = 5.9 Hz, 2H), 9.05 (dd, J = 9.3, 6.6 Hz, 1H), 8.98 (d, J = 9.1 Hz, 4H), 8.85 (t, J = 7.9 Hz, 2H), 8.20 (t, J = 6.7 Hz, 2H); ^{13}C NMR (101 MHz, DMSO-d_6) δ 158.4, 152.2, 151.9, 147.8, 146.8, 131.2, 129.3, 127.5; ^{31}P NMR (121 MHz, DMSO-d_6) δ −144.23 (sept, J = 711.4 Hz, 2P, PF_6). Spectral data matches those reported previously.[25]

4. Optimized Cartesian coordinates of all compounds (1)-(8)

(1)

31

C	0.00027693568292	1.20720294463976	3.37316059837057
C	0.00042997960585	0.00000002924569	4.05393070997509
C	0.00027697343714	-1.20720289498032	3.37316060009386
C	-0.00002812469336	-1.20320959380860	1.98146775868219
N	-0.00014816847470	0.00000000186432	1.36183421805055
C	-0.00002816196928	1.20320960629222	1.98146775819008
H	0.00038942903523	2.14172694785973	3.91204782922289
H	0.00066664754583	0.00000003381075	5.13533351160016
H	0.00038949883716	-2.14172690452131	3.91204779138805
Au	-0.00067868978215	0.00000001730492	-0.64093287459564
Cl	0.00607456284963	-0.00000022036656	-2.94482572248133
C	-0.00023592495485	-2.34482158880401	1.07468219205007
C	-0.00010816206624	-3.67010420351137	1.51349809589612
C	-0.00025374870395	-4.70232503920810	0.59138229002413
C	-0.00051585483201	-4.41972073287027	-0.76944708419744
C	-0.00064871261900	-3.10170409782023	-1.21534712980305
C	-0.00053128215231	-2.05597444138955	-0.31161513711770
H	0.00011955011651	-3.90438118837608	2.57041347400546
H	-0.00014998050154	-5.72852443941771	0.93308236053939
H	-0.00062097942307	-5.23119738139964	-1.48642092790115
H	-0.00086538420913	-2.88591922223438	-2.27534508423716
C	-0.00023595256039	2.34482160229858	1.07468220974200
C	-0.00010821479321	3.67010421513864	1.51349814564178
C	-0.00025374497494	4.70232506093306	0.59138233230906
C	-0.00051582286980	4.41972071674787	-0.76944705328487
C	-0.00064864496641	3.10170408356888	-1.21534711605578
C	-0.00053126894401	2.05597443462970	-0.31161511870278
H	0.00011944862709	3.90438117794958	2.57041353470098
H	-0.00015001172882	5.72852447423876	0.93308239237998
H	-0.00062090278257	5.23119739726744	-1.48642088116766
H	-0.00086528773562	2.88591920491823	-2.27534508177326

Energies (au)

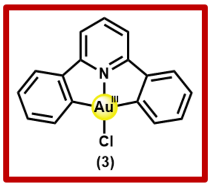

$E_{B97\text{-}3c}$ = -1305.065488915940
Thermal correction to $G_{298.15K}$ = 0.18293177

$E_{PBE0/def2\text{-}TZVPP}$ = -1304.436565052539

(2)

75

C	0.00051840852303	1.21058033278237	4.11016628672557
C	0.00076344718499	0.00000068446840	4.79646997028780
C	0.00051892704963	-1.21057926997600	4.11016679966791
C	-0.00002127570302	-1.18419312948379	2.72717687345580
N	-0.00033749185695	0.00000013793335	2.12188914197953
C	-0.00002172887479	1.18419361815836	2.72717639337016
H	0.00080229225520	2.16757085316139	4.61071077995311
H	0.00118573690844	0.00000093148124	5.87731783801528
H	0.00080326439820	-2.16756959188804	4.61071169715493
Au	-0.00077731139370	-0.00000010710900	0.12812365392649
Cl	-0.00141233814211	-0.00000025334207	-2.17847621014743
C	-0.00004880932322	-2.38260573081313	1.81258074541037
N	-0.00046565907428	-2.04905280279304	0.49449272389840
C	-0.00004950420089	2.38260592934413	1.81257993040727
N	-0.00046612279288	2.04905264312296	0.49449202911772
O	0.00044140971555	-3.51733288582141	2.27655413935681
O	0.00044015826377	3.51733320673507	2.27655301854800
C	-0.00016900338845	-3.03020337835747	-0.51654889541317
C	-1.22651481217105	-3.48123832374090	-1.02803817645896
C	-1.19990298760005	-4.42731189776072	-2.04408458663073
C	0.00026377570981	-4.90109473645028	-2.54662784066204
C	1.20022522604517	-4.42694430762578	-2.04392073887980
C	1.22642109893747	-3.48087166907237	-1.02787001152934
H	-2.13066488513817	-4.79752670872096	-2.45258041507552
H	0.00042814552905	-5.63664543813553	-3.34027420063182
H	2.13115536781344	-4.79684697815323	-2.45232129791840
C	-0.00016881121309	3.03020310705025	-0.51654965480546
C	-1.22651417927816	3.48123752564380	-1.02804033696635
C	-1.19990161900892	4.42731222755565	-2.04408562735121
C	0.00026547139322	4.90109620485527	-2.54662699275566
C	1.20022651892719	4.42694562517051	-2.04391908968575

Energies (au)

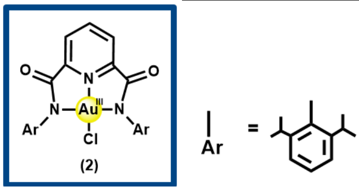

$E_{B97\text{-}3c}$ = -2113.878091418569
Thermal correction to $G_{298.15K}$ = 0.54611875

$E_{PBE0/def2\text{-}TZVPP}$ = -2112.942303585588

C	1.22642162948500	3.48087210857901	-1.02786923025752	
H	-2.13066321962531	4.79752685080136	-2.45258229852985	
H	0.00043040603037	5.63664780644200	-3.34027248390711	
H	2.13115693089162	4.79684896765189	-2.45231841356877	
C	2.54221173167771	2.95799806789150	-0.50679166361208	
C	3.35815746247530	4.06440974672204	0.15716061319939	
C	3.33027816619113	2.24505239294456	-1.60318048416469	
H	3.64823142362524	4.83067551982930	-0.56106898937221	
H	2.78781364211854	4.54711898168141	0.94846669878933	
H	4.27176311559484	3.65789251800881	0.59148428631703	
H	2.73486710030974	1.45762842480834	-2.06022881287176	
H	3.63288110066987	2.93425945844345	-2.39074791308147	
H	4.23523279880618	1.79701956500505	-1.19236951329646	
H	2.32002401385129	2.21736425555119	0.25984192902729	
C	-2.54248555175456	2.95865841500093	-0.50711507639105	
C	-3.35812858441443	4.06520655013923	0.15695950699728	
C	-3.33070191308792	2.24607453300413	-1.60365368874093	
H	-2.78769933733773	4.54757383991403	0.94841382161938	
H	-3.64785227850884	4.83170456166978	-0.56116582556035	
H	-4.27191767554640	3.65891923936707	0.59111154454284	
H	-3.63330490451214	2.93552853942058	-2.39100665804513	
H	-2.73536572928066	1.45873781638256	-2.06098437784753	
H	-4.23566831147226	1.79798972577012	-1.19293153938611	
H	-2.32051755185429	2.21785080727667	0.25942053231045	
C	2.54221155118009	-2.95799796200254	-0.50679292085754	
C	3.35815763309918	-4.06441017362600	0.15715796883242	
C	3.33027747192945	-2.24505130839134	-1.60318147382636	
H	2.78781419822650	-4.54712007595699	0.94846392180289	
H	3.64823124177178	-4.83067531988195	-0.56107245151538	
H	4.27176349572831	-3.65789329275584	0.59148154725793	
H	3.63288026562478	-2.93425765965905	-2.39074960207937	
H	2.73486611369349	-1.45762703408009	-2.06022888059081	
H	4.23523218668869	-1.79701868668697	-1.19237042016580	
H	2.32002426379376	-2.21736482537425	0.25984146645919	
C	-2.54248576125563	-2.95866054671573	-0.50711045717459	
C	-3.35812886109791	-4.06521044950725	0.15696099496095	
C	-3.33070254692328	-2.24607284977336	-1.60364629099336	
H	-3.64785330165460	-4.83170606492680	-0.56116660214045	
H	-2.78769946008175	-4.54758051378463	0.94841349388653	
H	-4.27191752528217	-3.65892410069927	0.59111477284442	
H	-2.73536630553349	-1.45873495363998	-2.06097486259520	
H	-3.63330629245232	-2.93552427302740	-2.39100123588318	
H	-4.23566849294073	-1.79798893218290	-1.19292211524807	
H	-2.32051704834088	-2.21785548785175	0.25942740732582	

(3)

31

C	-0.00000745140829	1.21251709879295	-3.36651078855993
C	-0.00001146662765	0.00000002514756	-4.04638584574464
C	-0.00000726702659	-1.21251706716815	-3.36651080641277
C	0.00000092437306	-1.20009990048035	-1.97843197308252
N	0.00000412352506	0.00000001000250	-1.36890415780439
C	0.00000076984425	1.20009992619694	-1.97843196572180
H	-0.00001067995350	2.14229206174386	-3.91411410219942
H	-0.00001787978183	-0.00000001097693	-5.12655320414348
H	-0.00001030265112	-2.14229200434805	-3.91411418828251
Au	0.00001824700920	-0.00000004228438	0.61860749791699
Cl	-0.00016830286217	0.00000021873328	2.89930780880979
C	0.00000647694106	-2.33776504990746	-1.05470431937543
C	0.00000323092020	-3.66538087154417	-1.43734506343866
C	0.00000723122318	-4.66517617448376	-0.47151862020997
C	0.00001413393614	-4.32029526774790	0.86790056552070
C	0.00001738672411	-2.98026810301259	1.22161523324968
N	0.00001401934097	-2.02847481796077	0.28505842289659
H	-0.00000285248088	-3.92481315160589	-2.48525780539675
H	0.00000456976017	-5.70318640876144	-0.77087596716457
H	0.00001718966848	-5.06835782209616	1.64641884041390
H	0.00002307606089	-2.64854940522613	2.24818796907174
C	0.00000633692227	2.33776502803875	-1.05470429740687
C	0.00000302738818	3.66538082535189	-1.43734505381565
C	0.00000718721246	4.66517613082586	-0.47151861546472
C	0.00001434108336	4.32029525005435	0.86790057342724
C	0.00001760359102	2.98026809958099	1.22161526899630
N	0.00001406847250	2.02847481081906	0.28505844186028
H	-0.00000322519592	3.92481309003392	-2.48525780963628
H	0.00000447700301	5.70318634745687	-0.77087599559012
H	0.00001755531339	5.06835781727879	1.64641883622878
H	0.00002345167500	2.64854935754655	2.24818797872138

Energies (au)

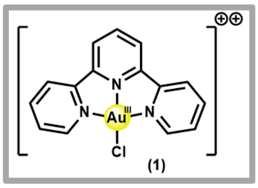

$E_{B97\text{-}3c}$ = -1337.798404156017
Thermal correction to $G_{298.15K}$ = 0.18498740

$E_{PBE0/def2\text{-}TZVPP}$ = -1337.164276691685

(4)

87

Coordinates from ORCA-job orca

C	2.92792604194148	0.38032929253072	1.68013732724634
C	3.16107018915171	0.28658668909941	3.03791105013458
H	3.98445013427785	0.81589744435560	3.49031030861320
C	2.34570632872689	-0.50378751465765	3.84398316940843
H	2.54526316781014	-0.57294182844396	4.90525135192162
C	1.27896128836235	-1.18821870967917	3.28532999221312
H	0.64162388766952	-1.80368163755669	3.90657366089219
C	1.03210804369155	-1.09185949288714	1.92382224821267
H	0.20574741238276	-1.64266683236073	1.49433631389160
C	1.85084762413072	-0.31889788895583	1.10365642800297
C	1.73159033455942	-0.19813202023870	-0.34202309588778
C	0.71547576184021	-0.74609417990767	-1.12110136864535
H	-0.08510697763627	-1.30973066671594	-0.65891096153046
C	0.71621710270061	-0.56721432124362	-2.49610044232126
H	-0.07785614114006	-0.99417815264342	-3.09486531898031
C	1.73311734073361	0.15728439041953	-3.10051587782267
H	1.73602375255792	0.29697866532279	-4.17421304499167
C	2.75557276055345	0.70833183995609	-2.33244563588000
H	3.54920548969970	1.26480045681713	-2.80747602930787
C	2.75932564192398	0.53516491455061	-0.95986054527451
C	5.49469667629662	2.08301108173482	1.76386960781482
C	7.25458951738788	2.25694260675923	3.16625169670021
H	8.21303921315971	1.99647569858167	3.57029533022460
C	6.37176634057531	3.21557757827717	3.51144516318960
H	6.41097939928289	3.97305343229932	4.26896303070040
C	7.46779792753298	0.64197754122779	1.29245188252703
C	7.18009482955702	-0.72174985072819	1.37825757589500
C	7.94682079009277	-1.58913183139289	0.60561335168325
H	7.74489241130703	-2.65017122338002	0.64554927074039
C	8.96002865403081	-1.11650169376002	-0.20602257066927
H	9.54319026985225	-1.80718259200796	-0.80057500279900
C	9.22676533669728	0.23959137369211	-0.26497168946923
H	10.01646747742074	0.59714773074318	-0.90998964095946
C	8.48617136183502	1.15363139021916	0.47526372121956
C	6.11890311395797	-1.27352499285314	2.29505941610222
H	5.52269515244345	-0.44247175543001	2.65310777214607
C	6.75133043115390	-1.93250898513701	3.51982215390565
H	7.35909937204452	-2.79149119882829	3.23666650565756
H	5.97792345244543	-2.28046405119401	4.20436673189843
H	7.39391780507849	-1.23673092462799	4.05902360053837
C	5.16432178241769	-2.22411906692449	1.58167135074950
H	4.76325338765283	-1.77425229565732	0.67704360271407
H	4.32506367425183	-2.46466758962693	2.23133012072506
H	5.65225010881020	-3.15887599683418	1.30754303531084
C	8.82117873758733	2.62726555763565	0.39712803436759

Energies (au)

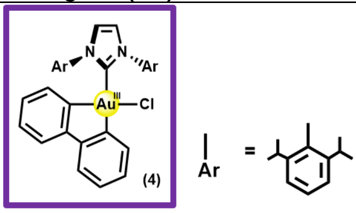

$E_{B97\text{-}3c}$ = -2217.640733708625
Thermal correction to $G_{298.15K}$ = 0.65766633

$E_{PBE0/def2\text{-}TZVPP}$ = -2216.634709195497

H	7.99086259725177	3.18603006031177	0.82357019138824
C	8.99624379323513	3.12327158919074	-1.03492125229742
H	9.10542627181298	4.20753732843645	-1.03752050238517
H	8.13526658943855	2.86416878038734	-1.64193032560384
H	9.88968242504572	2.71044432598025	-1.50147560656445
C	10.07387017653900	2.94157912417099	1.21729882744290
H	9.98036318656931	2.62674372153074	2.25488188163300
H	10.27671840809885	4.01227910771635	1.20817203533917
H	10.94319624735905	2.43356664674108	0.80108551436983
C	4.20323302975991	4.03801504793016	2.59111603154161
C	4.14536978821886	4.91271880534935	1.49266147812903
C	3.07348852010565	5.79626612890198	1.43657259504447
H	2.98810848629545	6.46929731179938	0.59717286636204
C	2.12446205437256	5.84138466247096	2.44179118208926
H	1.29758591401396	6.53574691929977	2.37390904251664
C	2.24410426129114	5.01568155579599	3.54157324972773
H	1.51483963797235	5.08094155909281	4.33596820905107
C	3.28692301080422	4.09869363583628	3.64770523445861
C	5.25133734776561	4.99630771357014	0.46652249696405
H	5.66914748209779	4.00572848647778	0.32163715952678
C	4.78666804194858	5.45185086716377	-0.90804810561175
H	3.95384466028437	4.84969977628692	-1.26219285677406
H	5.59828314144410	5.32753767787518	-1.62088546732347
H	4.49382073044720	6.50202061950382	-0.92117035743256
C	6.36914518635541	5.90146125972755	0.99044836242518
H	6.00895297025009	6.91942274878416	1.14008469613528
H	7.19059581197917	5.93557779573060	0.27556393578836
H	6.76776250722381	5.54609306399064	1.93945593618205
C	3.41390719237103	3.28454904504438	4.91524643614379
H	4.12643937861069	2.48148721046258	4.73785915628560
C	2.09536507414655	2.64644830936710	5.34268659640381
H	1.38746727265357	3.39199648649782	5.70107699704010
H	2.26558264739764	1.94801365393571	6.16100770806829
H	1.63162509311970	2.10183445965871	4.52664720689219
C	3.96639831360736	4.15187705871307	6.04907666016060
H	3.26792015463416	4.95037050964303	6.29558184753532
H	4.91225054318698	4.62249149143546	5.78664590562065
H	4.12276072486190	3.55486429856876	6.94713195676656
N	6.71378285693950	1.58371993600188	2.08406070575856
N	5.29460970517556	3.09806100436340	2.64624349047155
Cl	5.60478498022080	2.08192189088993	-1.44109616440617
Au	4.15790937828409	1.30530793481605	0.33525646432835

(5a)

Energies (au)

5

Au	-0.00000000186419	-0.00000000105475	0.00000000000000
Cl	2.34554138929313	0.00000000010047	0.00000000000000
Cl	-2.34554133644262	-0.00000001173789	0.00000000000000
Cl	-0.00000001957398	2.34554126781385	0.00000000000000
Cl	-0.00000003141234	-2.34554125512167	0.00000000000000

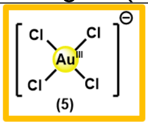

$E_{B97\text{-}3c}$ = -1976.827108628245
Thermal correction to $G_{298.15K}$ = -0.02601941

$E_{PBE0/def2\text{-}TZVPP}$ = -1976.190605185824

(5b)

Energies (au)

5

Au	0.00000000377443	-0.00000000362261	0.00000000000000
Br	2.48615277635825	-0.00000001918831	0.00000000000000
Br	-2.48615279514008	0.00000005360926	0.00000000000000
Br	-0.00000002889508	2.48615276534222	0.00000000000000
Br	0.00000004390249	-2.48615279614056	0.00000000000000

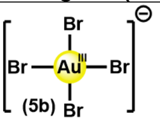

$E_{B97\text{-}3c}$ = -10436.615700389726
Thermal correction to $G_{298.15K}$ = -0.03216187

$E_{PBE0/def2\text{-}TZVPP}$ = -10431.407290523213

(6)

67

Au	0.00001117137827	-0.00000042309497	1.26116371471654
C	-0.00000793750854	-0.00000017811847	-0.70664492304410
N	-0.00001109859278	1.07115859210369	-1.53878487000777
C	-0.00001968311208	0.67614264824142	-2.86435047566694
C	-0.00001978860184	-0.67614228164178	-2.86435068912118
N	-0.00001176622715	-1.07115865268516	-1.53878522946195
H	-0.00002300145081	-1.38910925750693	-3.66679900781249
H	-0.00002272743140	1.38910989072691	-3.66679858675795
C	-0.00000330788350	-2.43019273346291	-1.08168268537054
C	1.22895184340373	-3.05434491112169	-0.85604754399757
C	1.20077021277460	-4.37211429218343	-0.41117873264464
C	0.00001614460947	-5.02561290906689	-0.19528085583042
C	-1.20074772675464	-4.37213293411240	-0.41118082353822
C	-1.22894940597387	-3.05436360777511	-0.85604861610212
H	2.13069167483929	-4.88835982675758	-0.21707601337336
H	0.00002375859720	-6.04869530256765	0.15635384190928
H	-2.13066146737093	-4.88839321349139	-0.21708033627883
C	-0.00000206414458	2.43019254166751	-1.08168189719958
C	1.22895325749823	3.05434518001023	-0.85604893072827
C	1.20077192426574	4.37211462636705	-0.41118027232058
C	0.00001798944585	5.02561291384507	-0.19528068471343
C	-1.20074600583073	4.37213214849240	-0.41117749510685
C	-1.22894795687789	3.05436282785284	-0.85604528462755
H	2.13069354568596	4.88836067830384	-0.21707965333030
H	0.00002585899123	6.04869537369179	0.15635384531956
H	-2.13065957585724	4.88839209189889	-0.21707531556146
C	-2.54426762417762	2.33157582938852	-1.02789304832790
C	-3.47168615130174	3.05380697843353	-2.00096262735197
H	-2.33726896108589	1.34977184344777	-1.45068257610469
C	-3.21203198319181	2.10319256116921	0.32695019600450
H	-3.77178324144259	4.03027665813398	-1.62359739095920
H	-2.99344855630620	3.20576544439116	-2.96797522131887
H	-4.37959689320283	2.47292649935429	-2.15981357080342
H	-2.55185286529400	1.56321194051382	1.00288948795374
H	-3.47605195458109	3.04705759859526	0.80228399490851
H	-4.12656451145205	1.52280681462273	0.20755832969422
C	2.54426107580787	2.33153697934585	-1.02789763692590
C	3.47169267782240	3.05375536645874	-2.00096428878672
C	3.21201970896870	2.10313784204144	0.32694579718318
H	2.33724620566816	1.34973765046945	-1.45069019182742
H	2.99345848720518	3.20572468946565	-2.96797686022277
H	3.77180581126207	4.03021892143187	-1.62359596480949
H	4.37959380790745	2.47286011745703	-2.15981614047691
H	3.47605548086055	3.04699677576586	0.80228296806997
H	2.55183016717128	1.56316664700958	1.00288251364147
H	4.12654228989178	1.52273645322980	0.20755339128368
C	-2.54426913193799	-2.33157720567172	-1.02789895763370
C	-3.47168588805545	-3.05380934468954	-2.00096949661064
C	-3.21203566958307	-2.10319334329658	0.32694313251303
H	-2.33727024198012	-1.34977341044497	-1.45068880460962
H	-2.99344668730743	-3.20576839423792	-2.96798119758420
H	-3.77178335206080	-4.03027882645582	-1.62360404999949
H	-4.37959652495719	-2.47292921601240	-2.15982235883139
H	-3.47605586111548	-3.04705819519506	0.80227717786274
H	-2.55185782767068	-1.56321196345331	1.00288306196703
H	-4.12656829998179	-1.52280810729031	0.20754948563851
C	2.54425964410316	-2.33153597856334	-1.02789354473951
C	3.47169453854390	-3.05375483126058	-2.00095673938505
H	2.33724522188303	-1.34973731348357	-1.45068787000777
C	3.21201441194807	-2.10313467759044	0.32695144252902
H	3.77180769641402	-4.03021753391981	-1.62358624816612
H	2.99346312110432	-3.20572600136708	-2.96797038632377
H	4.37959549388054	-2.47285885709933	-2.15980698848851
H	2.55182261339388	-1.56316308214264	1.00288564174145
H	3.47604952814934	-3.04699282173051	0.80229053372177
H	4.12653690029216	-1.52273275687036	0.20756081026406
Cl	0.00003347653636	-0.00000073956554	3.56135635372934

Energies (au)

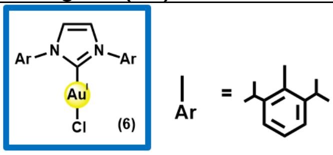

$E_{B97\text{-}3c}$ = -1755.807498477103

Thermal correction to $G_{298.15K}$ = 0.50565322

$E_{PBE0/def2\text{-}TZVPP}$ = -1754.983195072371

(7)

36

P	0.00080564921558	-0.00021945332308	0.61901379574888
C	1.67039645527419	-0.00782913800741	1.34658440013854
C	2.66021076982668	0.75770935500387	0.73211492009368
C	3.93067214691904	0.81809409321918	1.28059554123492
C	4.22471901123290	0.10759029307406	2.43719270048714
C	3.24393287743318	-0.66221928981312	3.04568553968841
C	1.96770254141620	-0.72076627461561	2.50463643561217
H	2.43447944451890	1.29280269001467	-0.18106506284946
H	4.69569927758737	1.41020089628377	0.79727872391567

Energies (au)

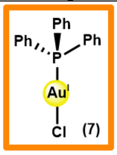

$E_{B97\text{-}3c}$ = -1632.197223998684

H	5.21994720384704	0.14873176891128	2.85932910964138
H	3.47218271018817	-1.22356133392586	3.94163890057517
H	1.20781185510677	-1.32658064016986	2.97790455056107
C	-0.84152340010169	-1.44236057918459	1.34525920184779
C	-0.67225963469962	-2.68293742425363	0.73224745715737
C	-1.25715060298555	-3.81264104302392	1.28018304791421
C	-2.02244821643566	-3.71080087246742	2.43471106008831
C	-2.19986924094202	-2.47593270171591	3.04170803988590
C	-1.61064924447637	-1.34214602383183	2.50114991542178
H	-0.09355375191653	-2.75619204278067	-0.17931284470637
H	-1.12596481325026	-4.77169426979609	0.79804508767658
H	-2.48583304092745	-4.59276216765811	2.85636507439672
H	-2.80243762122566	-2.39195861871123	3.93602160844187
H	-1.75626889745460	-0.38055813540961	2.97287914268593
C	-0.82767580126944	1.44953476811843	1.34617517245735
C	-1.98936847065114	1.92016101886990	0.73594496787819
C	-2.67652518669747	2.99003504132747	1.28525019015332
C	-2.20414666458644	3.60358572280435	2.43824300827667
C	-1.04328672743856	3.14295561793923	3.04239324983172
C	-0.35483300226748	2.06714078973746	2.50059227304329
H	-2.34363637766038	1.45411119328250	-0.17428616072664
H	-3.57487470835234	3.35346548526045	0.80531382536205
H	-2.73724131142955	4.44474059352503	2.86089875864882
H	-0.66818943798337	3.62406446745433	3.93557586440486
H	0.55248758616033	1.71441876342073	2.97039542837825
Au	0.00242476206288	0.00052436672102	-1.62042174674205
Cl	0.00426386196236	0.00129308372023	-3.92205492953419

Thermal correction to $G_{298.15K}$ = 0.22400618

$E_{PBE0/def2\text{-}TZVPP}$ = -1631.503581082626

(8)

17

C	0.00153977719479	1.21257526491189	3.99701191451485
C	0.00200954285696	-0.00000113622400	4.68249777237775
C	0.00158288060983	-1.21257742408096	3.99700946752431
C	0.00068482496456	-1.18478777214297	2.61445430356538
N	0.00022311283029	0.00000127366466	2.01317564976232
C	0.00063678866141	1.18478882845741	2.61445614422435
H	0.00188551519942	2.16618196434513	4.50443182077611
H	0.00277903463780	-0.00000204157335	5.76305787495286
H	0.00196753737848	-2.16618440139213	4.50442849072955
Au	0.00009672536847	0.00000353103062	0.03294244225209
Cl	-0.00005182124124	0.00000062214547	-2.25022125243277
C	0.00041222718067	-2.35675524085315	1.65587061485642
O	0.00017253193348	-2.04714092468472	0.36634064273509
C	0.00033545341800	2.35675919596558	1.65587371234199
O	0.00007166901403	2.04714347566699	0.36634888502808
O	0.00040653519627	-3.50017328561553	2.05644534457005
O	0.00033535479678	3.50017445437908	2.05645986422154

Energies (au)

(8)

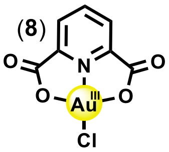

$E_{B97\text{-}3c}$ = -1220.151326748549

Thermal correction to $G_{298.15K}$ = 0.05607128

$E_{PBE0/def2\text{-}TZVPP}$ = -1219.591475635933

Oxygen-based ligands

In order to increase the generalizability of our findings, we include here the ONO-pincer gold(III) chloride complex, (pyridine-2,6-dicarboxylato)$Au^{III}Cl$, **(8)**. This complex also exhibits a strong whiteline absorption feature in its Au L_3-edge XANEs spectrum (Figure S3), as does its NNN-pincer analogue, **(2)**, (Figure S1).

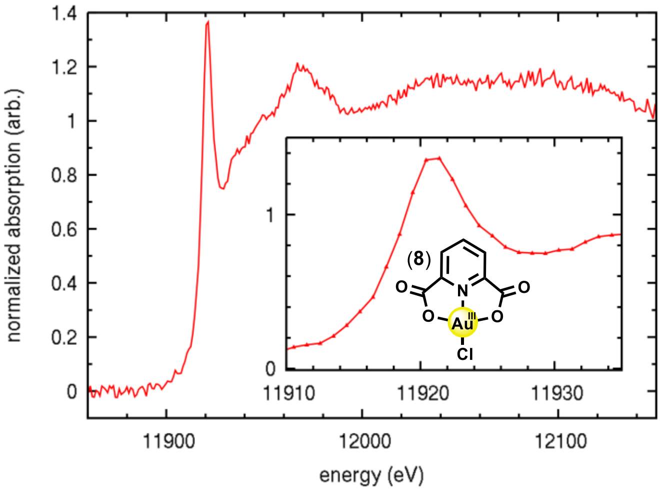

Figure S3: Normalized Au L_3-edge XAS spectra of (pyridine-2,6-dicarboxylato)gold(III) chloride, (**8**). The inset shows the edge region.

Intuitively, one may expect a decrease in the electron-sharing nature of the Au-O bonds, as compared to their Au-N counterparts. The IAO partial charge distributions of these σ-bonds are in line with these expectations: Au(0.39)O(1.53) and Au(0.45)N(1.45) (Tables S6 and 1, respectively). Furthermore, the electron density recovered by the metal, $\boldsymbol{\Sigma q_{\sigma\text{-}IBO}(Au)}$, is only 1.71 for **(8)** (Table S6), while it is greater (1.78) for **(2)** (Table 1). Finally, the same effect is reflected in the smaller difference between the $\boldsymbol{\Delta E_{orb}}$ terms of the d^8 *and* d^{10} configurations in the EDA (Table S2): $\boldsymbol{\Delta\Delta E_{orb:d8\text{-}10}(2,8)}$ =*(-567, -431)* kcal mol^{-1}.

Table S6: The IBOs of (pyridine-2,6-dicarboxylato)gold(III) chloride, (**8**), showing its intrinsic-d^8 configuration (top) and metal-ligand σ-bonds (bottom). Calculated with (PBE0/def2-TZVPP//B97-3c).

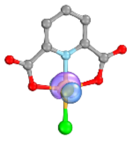	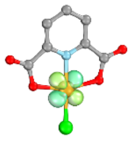	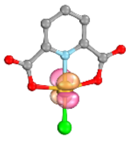	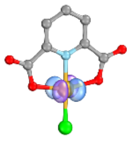
Au(2.00)	Au(1.99)	Au(1.98)	Au(2.00)
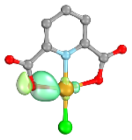	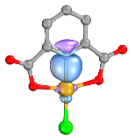	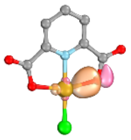	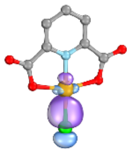
Au(0.39) O(1.53)	Au(0.36) N(1.54)	Au(0.39) O(1.53)	Au(0.57) Cl(1.40)

$\boldsymbol{\Sigma q_{\sigma\text{-}IBO}(Au) = 1.71}$

References

[1] H. A. Rowland, *The London, Edinburgh, and Dublin Philosophical Magazine and Journal of Science* **1883**, *16*, 197-210.

[2] H. H. Johann, *Zeitschrift für Physik* **1931**, *69*, 185-206.

[3] B. Ravel, M. Newville, *Journal of Synchrotron Radiation* **2005**, *12*, 537-541.

[4] a) F. Neese, *WIREs: Comput. Mol. Sci.* **2012**, *2*, 73-78; b) F. Neese, *WIREs: Comput. Mol. Sci.* **2018**, *8*, e1327.

[5] J. G. Brandenburg, C. Bannwarth, A. Hansen, S. Grimme, *J. Chem. Phys.* **2018**, *148*, 064104.

[6] A. D. Becke, *J. Chem. Phys.* **1997**, *107*, 8554-8560.

[7] F. Weigend, R. Ahlrichs, *Phys. Chem. Chem. Phys.* **2005**, *7*, 3297-3305.

[8] a) D. Andrae, U. Häußermann, M. Dolg, H. Stoll, H. Preuß, *Theor. Chim. Acta* **1990**, *77*, 123-141; b) R. Flores-Moreno, R. J. Alvarez-Mendez, A. Vela, A. M. Köster, *J. Comput. Chem.* **2006**, *27*, 1009-1019.

[9] a) S. Grimme, J. Antony, S. Ehrlich, H. Krieg, *J. Chem. Phys.* **2010**, *132*, 154104; b) S. Grimme, A. Hansen, J. G. Brandenburg, C. Bannwarth, *Chem. Rev.* **2016**, *116*, 5105-5154.

[10] G. Knizia, *J. Chem. Theory Comput.* **2013**, *9*, 4834-4843.

[11] G. Knizia, J. E. M. N. Klein, *Angew. Chem. Int. Ed.* **2015**, *54*, 5518-5522.

[12] C. Adamo, M. Cossi, V. Barone, *J. Mol. Struct.: THEOCHEM* **1999**, *493*, 145-157.

[13] F. Neese, F. Wennmohs, A. Hansen, U. Becker, *Chem. Phys.* **2009**, *356*, 98-109.

[14] a) K. Kitaura, K. Morokuma, *Int. J. Quantum Chem* **1976**, *10*, 325-340; b) T. Ziegler, A. Rauk, *Inorg. Chem.* **1979**, *18*, 1755-1759; c) T. Ziegler, A. Rauk, *Inorg. Chem.* **1979**, *18*, 1558-1565.

[15] G. te Velde, F. M. Bickelhaupt, E. J. Baerends, C. Fonseca Guerra, S. J. A. van Gisbergen, J. G. Snijders, T. Ziegler, *J. Comput. Chem.* **2001**, *22*, 931-967.

[16] E. Van Lenthe, E. J. Baerends, *J. Comput. Chem.* **2003**, *24*, 1142-1156.

[17] a) E. V. Lenthe, E. J. Baerends, J. G. Snijders, *J. Chem. Phys.* **1993**, *99*, 4597-4610; b) E. Van Lenthe, E. J. Baerends, J. G. Snijders, *J. Chem. Phys.* **1994**, *101*, 9783-9792; c) E. Van Lenthe, J. G. Snijders, E. J. Baerends, *J. Chem. Phys.* **1996**, *105*, 6505-6516; d) E. Van Lenthe, R. van Leeuwen, E. J. Baerends, J. G. Snijders, *Int. J. Quantum Chem* **1996**, *57*, 281-293; e) E. Van Lenthe, A. Ehlers, E.-J. Baerends, *J. Chem. Phys.* **1999**, *110*, 8943-8953.

[18] a) S. F. Boys, *Reviews of Modern Physics* **1960**, *32*, 296-299; b) J. M. Foster, S. F. Boys, *Reviews of Modern Physics* **1960**, *32*, 300-302.

[19] C. Edmiston, K. Ruedenberg, *Reviews of Modern Physics* **1963**, *35*, 457-464.

[20] J. Pipek, P. G. Mezey, *J. Chem. Phys.* **1989**, *90*, 4916-4926.

[21] J. S. Steen, G. Knizia, J. E. M. N. Klein, *Angew. Chem. Int. Ed.* **2019**, *58*, 13133-13139.

[22] P. Pyykkö, *Angew. Chem. Int. Ed.* **2004**, *43*, 4412-4456.

[23] a) K.-H. Wong, K.-K. Cheung, M. C.-W. Chan, C.-M. Che, *Organometallics* **1998**, *17*, 3505-3511; b) L. S. Hollis, S. J. Lippard, *J. Am. Chem. Soc.* **1983**, *105*, 4293-4299; c) S. Engbers, E. A. Trifonova, K. M. Hess, F. Vries, J. E. M. N. Klein, *Eur. J. Inorg. Chem.* **2021**, *2021*, 3561-3564; d) M. W. Johnson, A. G. Dipasquale, R. G. Bergman, F. D. Toste, *Organometallics* **2014**, *33*, 4169-4172; e) C.-Y. Wu, T. Horibe, C. B. Jacobsen, F. D. Toste, *Nature* **2015**, *517*, 449-454; f) Z.-T. Yu, X.-L. Liu, Y.-J. Yuan, Y.-H. Li, G.-H. Chen, Z.-G. Zou, *Dalton Trans.* **2016**, *45*, 17223-17232; g) M. Stollenz, D. Taher, N. Bhuvanesh, J. H. Reibenspies, Z. Baranová, J. A. Gladysz, *Chem. Commun.* **2015**, *51*, 16053-16056.

[24] U. Sampath, W. C. Putnam, T. A. Osiek, S. Touami, J. Xie, D. Cohen, A. Cagnolini, P. Droege, D. Klug, C. L. Barnes, A. Modak, J. K. Bashkin, S. S. Jurisson, *J. Chem. Soc., Dalton Trans.* **1999**, 2049-2058.

[25] K. Czerwińska, M. Golec, M. Skonieczna, J. Palion-Gazda, D. Zygadło, A. Szlapa-Kula, S. Krompiec, B. Machura, A. Szurko, *Dalton Trans.* **2017**, *46*, 3381-3392.
